# Green Tea Extract Containing Epigallocatechin-3-Gallate Facilitates Bone Formation and Mineralization by Alleviating Iron-Overload-Induced Oxidative Stress in Human Osteoblast-like (MG-63) Cells

**DOI:** 10.3390/antiox14070874

**Published:** 2025-07-17

**Authors:** Honghong Xu, Orawan Khantamat, Woranontee Korsieporn, Narisara Paradee, Jin Li, Yanping Zhong, Somdet Srichairatanakool, Pimpisid Koonyosying

**Affiliations:** 1 Department of Biochemistry, Faculty of Medicine, Chiang Mai University, Chiang Mai 50200, Thailand; honghong_xu@cmu.ac.th (H.X.); orawan.kh@cmu.ac.th (O.K.); woranontee.k@cmu.ac.th (W.K.); narisara.p@cmu.ac.th (N.P.); jin_lijin@ymun.edu.cn (J.L.); yanping_z@cmu.ac.th (Y.Z.); somdet.s@cmu.ac.th (S.S.); 2 School of Biochemistry, School of Basic Medical Science, Youjiang Medical University for Nationalities, Baise 533000, China; 3 School of Medical Technology and Artificial Intelligence, Youjiang Medical University for Nationalities, Baise 533000, China

**Keywords:** green tea, EGCG, iron overload, oxidative stress, bone formation, bone mineralization

## Abstract

Secondary iron overload exacerbates osteoporosis by elevating reactive oxygen species (ROS), which suppress osteoblast function and enhance osteoclast activity, disrupting bone remodeling. Reducing iron overload and oxidative stress may improve bone health. Epigallocatechin-3-gallate (EGCG), the main bioactive compound in green tea extract (GTE), is recognized for its antioxidant and iron-chelating properties. This study examined the effect of GTE on bone formation and mineralization in iron-overloaded human osteoblast-like MG-63 cells. An iron-overloaded model was established using ferric ammonium citrate (FAC), followed by treatment with GTE, deferiprone (DFP), or their combination. GTE significantly reduced intracellular iron, ROS levels, and lipid peroxidation while upregulating the osteogenic marker BGLAP, the anti-resorptive marker OPG, and osteogenic mineralization, indicating restored bone health. These results suggest that EGCG-containing GTE mitigates iron-induced oxidative stress and promotes osteogenesis, highlighting its potential as a natural therapeutic supplement for managing iron-overload-associated osteoporosis.

## 1. Introduction

Several blood disorders, including β-thalassemia, myeloproliferative disorders, and sickle cell anemia, suffer from secondary iron overload caused by excessive iron absorption from frequent blood transfusions [1]. Iron overload ultimately results in systemic complications, including iron accumulation in the heart, liver, bone marrow, and glandular tissue [2]. Bone loss can be influenced by iron overload, arising from imbalances in bone metabolism, often driven by oxidative stress and excessive iron accumulation [3]. A high iron level induces reactive oxygen species (ROS) formation through the redox reaction of iron. ROS have a negative impact on osteoblast function while increasing osteoclast activity, which disturbs bone remodeling processes [4]. Additionally, excessive iron accumulation in bone tissue intensifies oxidative stress, weakening bone structures and heightening fracture possibilities. These variables lead to the elevated incidence of osteoporosis and other bone-related disorders in individuals with hematological diseases [5]. Given the critical role of oxidative stress and iron overload in bone loss, targeted interventions are essential to mitigate these effects and promote bone health.

Green tea (*Camellia sinesis*) extract (GTE), a traditional medicinal and dietary substance, is rich in a variety of polyphenolic compounds, including catechin (C), epicatechin-3-gallate (ECG), epigallocatechin (EGC), and epigallocatechin-3-gallate (EGCG) [6]. Among these, EGCG is well-regarded for its powerful iron-chelating capacity attributed to the presence of multiple hydroxyl groups and gallate moieties that can bind iron ions and inhibit iron-induced oxidative damage [7]. T. Petiwathayakorn et al. reported that EGCG functions as a natural iron chelator, effectively reducing iron levels and improving thrombosis in β-thalassemia patients [8]. Rasaei et al. demonstrated that GTE reduces oxidative stress and exerts alleviating effects in individuals with pre-existing inflammatory conditions [9]. Clinical studies on bone-related research indicate that GTE may help to prevent bone loss in postmenopausal women by reducing oxidative stress [10]. A recent study demonstrated that EGCG positively promotes osteogenic differentiation in human periodontal ligament cells [11]. In addition, GTE exhibits a wide range of biological activities, including anti-inflammatory effects, neuroprotection, and the modulation of lipid metabolism [12,13,14,15]. Despite its well-documented antioxidant and iron-chelating properties in various pathological conditions, the potential of GTE to promote bone formation and mineralization in iron-overload-induced skeletal damage has not been investigated. This study hypothesizes that the multifaceted properties of GTE, particularly its iron-chelating and antioxidant capabilities, may reduce iron-overload-induced oxidative stress, thereby enhancing osteoblast function and promoting bone formation and mineralization to mitigate bone loss in pathological iron-overload conditions.

## 2. Materials and Methods

### 2.1. Materials and Reagents

Human osteoblast-like MG-63 cells (CRL-1427, ATCC, Manassas, VA, USA) were obtained from the Thailand Excellence Center for Tissue Engineering and Stem Cells, Department of Biochemistry, Faculty of Medicine, Chiang Mai University, Thailand. Deferiprone (DFP) was purchased from (GPO-L-ONE^®^, Government Pharmaceutical Organization, Bangkok, Thailand). Ammonium ferric citrate (FAC) (#PCT0107, Himedia, Chester County PA, USA), Ferro-orange (F374-10, Dojindo, Rockville, MD, USA), and L-ascorbic acid (KA79, Kemaus, Cherrybrook, N.S.W., Australia) were also obtained. 3-(4, 5-dimethylthiazol-2-yl)-2, 5-diphenyl tetrazolium bromide (MTT) (M2003), L-ascorbic acid (A92902), β-glycerophosphate disodium salt hydrate (G9422), 2′,7′-dichlorodihydrofluorescein diacetate (DCFH-DA) (D6883), Alizarin red S (A5533), paraformaldehyde (158127), and KMnO_4_ (223468) were purchased from Sigma (St. Louis, MO, USA). Sandwich ELISA kits for human bone gamma-carboxyglutamate protein (BGLAP, or osteocalcin; abx252820) and human osteoprotegerin (OPG; abx152605) were purchased from the Abbexa company (Cambridge, UK).

### 2.2. Extraction and Analysis of Green Tea

Fresh tea leaves (*Camellia sinensis*) were collected from the Doi Angkhang tea fields in the Fang District, Chiang Mai, Thailand, and authenticated by Wannaree Charoensup, a botanist at the Herbarium of the Faculty of Pharmacy at Chiang Mai University (Voucher number: 0023404). To inactivate polyphenol oxidase (PPO) activity, the leaves were dried using a microwave oven (800 Watts, 3 min, and 120 °C). The dried leaves were blended and then extracted with deionized water at 80 °C (2 g/20 mL) for 10 min, followed by filtration. The filtrated solution was freeze-dried to obtain a green tea extract (GTE) powder. The EGCG content in GTE was determined using reverse-phase HPLC/DAD (Figure S1).

### 2.3. Cell Culture

Osteoblast-like MG-63 cells were cultured in Dulbecco’s Modified Eagle Medium (DMEM) supplemented with 10% fetal bovine serum (FBS), 100 U/mL penicillin, and 100 µg/mL streptomycin. The cells were incubated at 37 °C in a humidified atmosphere containing 5% CO_2_.

### 2.4. Cell Viability Assay

Cell viability was measured using the 3-(4,5-dimethylthiazol-2-yl)-2,5-diphenyl tetrazolium bromide (MTT) assay. MG-63 cells were treated with various concentrations of FAC (0–200 µM) and GTE (0–20 µM EGCG equivalent) for 24 and 48 h. The MTT solution (5 mg/mL) was added to 96-well plates and incubated for 4 h at 37 °C, followed by dissolution in DMSO. The absorbance of the formazan crystal in the cell supernatant was measured at 570 nm with a microplate reader.

### 2.5. Iron Mobilization Assay

The intracellular iron content was measured using a ferrozine colorimetric assay, following the research of Koonyosying [16], and a Ferro-orange fluorochrome. MG-63 cells were seeded at a density of 1 × 10^5^ cells per well in a 24-well plate and exposed to 150 µM FAC for 6 h to induce iron overload, followed by treatment with DFP (5 µM and 10 µM), GTE (5 µM and 10 µM EGCG equivalent), or their combination for a further 2 to 10 h. After treatment, the cells were lysed with 200 mM NaOH overnight and neutralized with 10 mM HCl. The lysate was incubated with an iron-releasing solution (1.4 M HCl and 4.5% KMnO_4_, 1:1) at room temperature for 2 h. A chromogenic reagent containing ferrozine (6.5 mM), neocuproine (6.5 mM), ammonium acetate (2.5 M), and L-ascorbic acid (1 M) was added and incubated for 30 min. The absorbance was measured at 562 nm. Finally, the iron content was normalized to the protein concentration. For the intracellular ferrous iron level using the Ferro-orange fluorochrome, 1 × 10^5^ cells were washed with PBS after iron induction and treatment. Then, the washed cells were incubated with Ferro-orange (1 µM) for 30 min at 37 °C. Fluorescence intensity (FI) was measured using a fluorescence microscope, and Ferro-orange absorbance was recorded at 570 nm with a microplate reader.

### 2.6. Measurement of Lipid Peroxidation

Lipid peroxidation was assessed by measuring the levels of the lipid peroxidation product, malondialdehyde (MDA). Next, 1 × 10^5^ MG-63 cells were induced with FAC (150 µM) for 6 h, followed by a treatment of 10 mg/mL vitamin E, DFP (5 µM and 10 µM), GTE (5 µM and 10 µM EGCG equivalent), or their combination for 8 h. Then, the cells were lysed by ultrasonication and administered with 1% meta-phosphoric acid (MPA) and 0.67% (*w*/*v*) thiobarbituric acid (TBA) at 95 °C for 1 h. After cooling, n-butanol was added to the samples and recorded at 532 nm with a microplate reader.

### 2.7. Reactive Oxygen Species (ROS) Measurement

Intracellular ROS levels were measured using a 2′,7′-dichlorodihydrofluorescein diacetate (DCFH-DA) probe. We treated 1 × 10^5^ MG-63 cells with FAC (150 µM) for 6 h to induce iron overload, followed by a treatment with DFP (5 µM and 10 µM), GTE (5 µM and 10 µM EGCG equivalent), or their combination for 8 h. The cells were then incubated with DCFH-DA (10 µM) for 30 min at 37 °C. FI was measured using a fluorescence microscope, images were analyzed using ImageJ 1.54 software, and absorbance was measured at 532 nm by a microplate reader.

### 2.8. Bone Formation Markers’ Determination

The levels of the bone formation markers bone gamma-carboxyglutamate protein (BGLAP, or osteocalcin) and osteoprotegerin (OPG) were quantified following the protocols of the sandwich enzyme-linked immunosorbent assay (ELISA) kits purchased from the Abbexa company.

### 2.9. Bone Mineralization Assessment

To assess the impact of GTE on bone mineralization, 1 × 10^5^ MG-63 cells were cul-tured in osteoblast differentiation medium containing ascorbic acid (50 µg/mL) and β-glycerophosphate (10 mM). The cells were treated with 150 µM FAC to induce iron overload, followed by treatment with GTE (5 µM, 10 µM EGCG equivalent), DFP (5 µM, 10 µM), or the combination for 6 days. The cells were fixed with 4% paraformaldehyde for 30 minutes at 37 °C, and stained with 0.1% Alizarin Red S (pH 8.3) for a further 30 min at 37 °C. Bone nodules were observed and photographed under a microscope. The absorbance was measured at 570 nm using a microplate reader.

### 2.10. Statistical Analysis

All experiments were independently repeated at least three times, with a minimum of three samples per group (*n* = 3) in each experiment, to ensure reproducibility and accuracy. Data were expressed as the mean ± standard error of the mean (SEM) to reflect the precision of the mean estimates for group comparisons as the SEM is particularly suitable for small-sample in vitro studies assessing treatment effects. The statistical significance was determined by a one-way ANOVA followed by Tukey’s multiple comparison test using GraphPad Prism software (Version 9.5), with *p* < 0.05 (*p* < 0.01, *p* < 0.001, and *p* < 0.0001) indicating significance. Prior to the one-way ANOVA, data distribution was confirmed using the Shapiro–Wilk test, and the homogeneity of variance was verified using Levene’s test, ensuring the appropriateness of the parametric analysis.

## 3. Results

### 3.1. Effects of GTE and FAC on MG-63 Cell Viability

**Figure 1:**
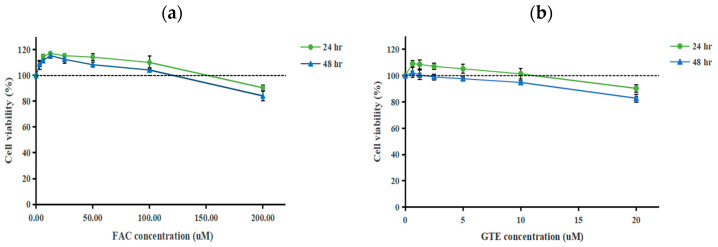
The viability of MG-63 cells after incubation of FAC (0–200 µM) for 24 and 48 h (**a**) and GTE (0–20 µM EGCG equivalent) for 24 and 48 h (**b**). FAC: ferric ammonium citrate; GTE: green tea extract; MTT: 3-(4,5-dimethylthiazol-2-yl)-2,5-diphenyl tetrazolium. The results are expressed as the mean ± SEM (*n* = 6) from three independent experiments, each with duplicate measurements.

MTT was used to monitor MG-63 cell survival under different concentrations of FAC and GTE at 24 h and 48 h. FAC at low concentrations (0–12.5 µM) slightly increased MG-63 cell viability over 24 and 48 h (Figure 1a). Thereafter, FAC at 12.5–100 µM presented a lower cell viability, but the percentage of cell viability was still above 100%. When the concentration was over 100 µM, FAC markedly diminished cell viability to around 80% in both the 24 h and 48 h experiments. Concurrently, the treatment of MG-63 cells with GTE (0–10 µM EGCG equivalent) (Figure 1b) maintained a cell viability level greater than 90%. Nevertheless, cell viability significantly declined to approximately 80% at 20 µM. Therefore, GTE at 5 µM and 10 µM EGCG equivalents was selected for further study to avoid potential cytotoxicity at higher doses.

### 3.2. GTE Effect on Intracellular Iron and Iron Mobilization

**Figure 2:**
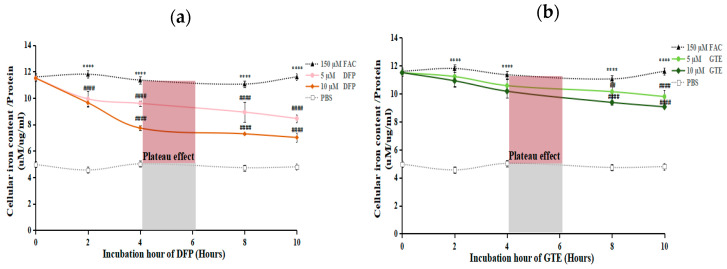

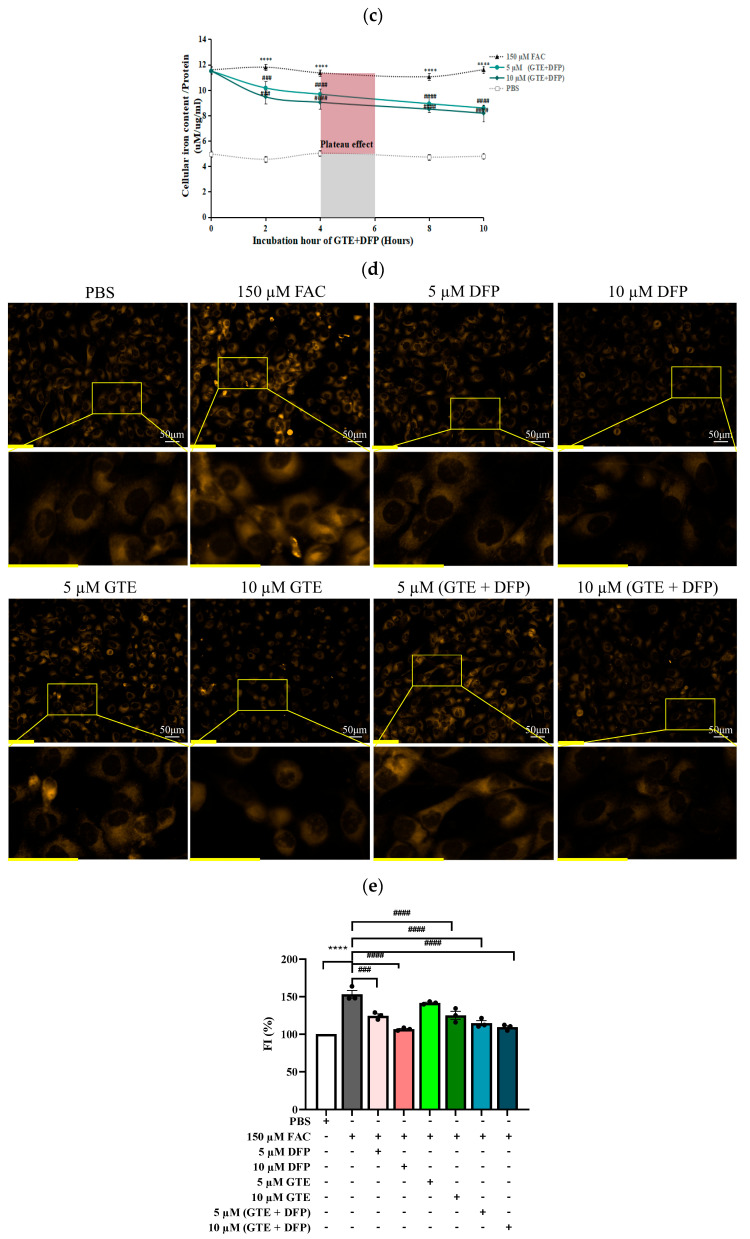
Iron concentration variations and iron-mobilizing activity of GTE in MG-63 cells. MG-63 cells with or without iron overload were treated with 5 µM and 10 µM DFP for 0–10 h (**a**). MG-63 cells with or without iron overload were treated with GTE (5 µM and 10 µM EGCG equivalent) for 0–10 h (**b**). MG-63 cells with or without iron overload were treated with 5 µM and 10 µM DFP, and GTE (EGCG equivalent) combination therapy, for 0–10 h (**c**). The Ferro-Orange fluorogenic probe was used to observe intracellular labile iron levels in MG-63 cells treated with 5 µM or 10 µM DFP, GTE (containing EGCG equivalents), or their combination for 8 h, with or without prior induction by 150 µM FAC for 6 h (under 200x magnification) (**d**). Statistics of iron content in MG-63 cells (**e**). Data are expressed as mean ± SEM (*n* = 3). Accordingly, **** *p* < 0.0001 when compared with PBS control. ^##^ *p* < 0.01, ^###^ *p* < 0.001, and ^####^ *p* < 0.001 when compared with FAC. FI: fluorescence intensity; FAC: ferric ammonium citrate; DFP: deferiprone; GTE: green tea extract.

According to the ferrozine assay, MG-63 cells loaded with 150 µM FAC showed a significant 2.4-fold increase (*p* < 0.0001) in total intracellular iron levels compared with non-treated (PBS) cells (Figure 2). DFP (5 µM and 10 µM), GTE (5 µM and 10 µM EGCG equivalent), and their combination resulted in a time-dependent reduction in intracellular iron content from 2 to 4 h. However, a plateau effect was observed after 4 h, indicating saturation of chelation efficacy, particularly in the 10 µM DFP sample, where intracellular iron levels stabilized without a significant further decline (Figure 2a). In contrast, 5 µM and 10 µM GTE alone and the combined therapy displayed a continued gradual decrease after the plateau effect period (Figure 2b,c). In addition, the 10 µM DFP and 10 µM GTE treatments, either alone or in combination, were more efficacious in mobilizing intracellular iron compared with the other treatments.

Consistently, fluorescence microscopy employing the Ferro-Orange fluorochrome to measure the ferrous content revealed significantly higher fluorescence intensity (FI) in MG-63 cells after being induced with FAC for 6 h (*p* < 0.0001) compared with the non-loading group. Similarly, FI was notably reduced in MG-63 cells following treatments with DFP and GTE alone, and their combination, after 8 h of incubation (Figure 2d,e), indicating that both treatments effectively decreased the amount of iron in the iron-overloaded MG-63 cells. Interestingly, the combination of DFP and GTE reduced the iron content. However, it did not provide an additive or synergistic effect in mobilizing iron compared with DFP and GTE alone.

### 3.3. Inhibition of Lipid Peroxidation and Antioxidant Properties of GTE

**Figure 3:**
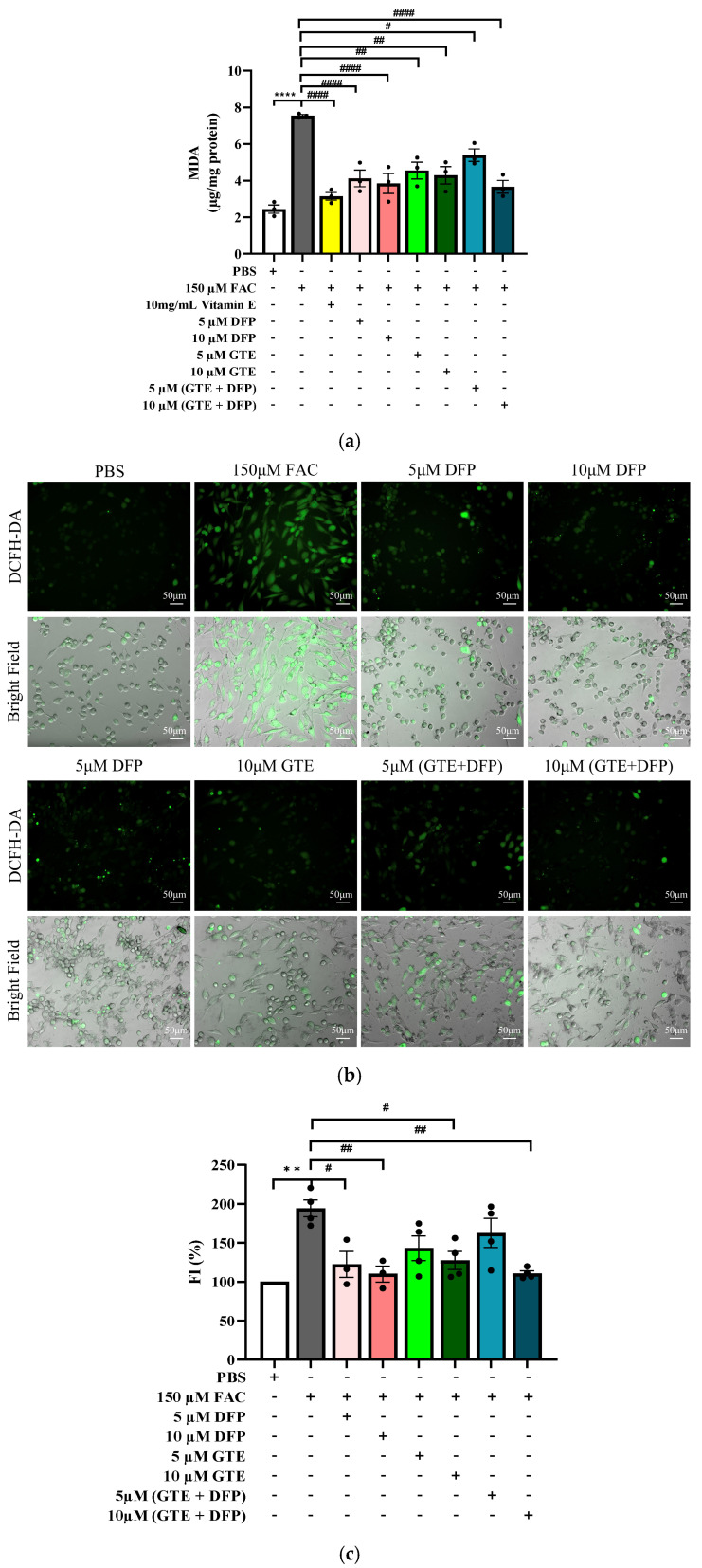
Inhibition of lipid peroxidation and antioxidant activity in MG-63 cells. MDA levels in MG-63 cells treated with 5 µM and 10 µM DFP, GTE (EGCG equivalent), and their combination with or without iron overload (**a**). DCF fluorescence in MG-63 cells treated with 5 µM and 10 µM DFP, GTE (EGCG equivalent), and their combination with or without iron overload (under 200x magnification) (**b**). Statistical results for the percentage of DCF fluorescence (**c**). Data are expressed as mean ± SEM (*n* = 3). Accordingly, ** *p* < 0.01 and **** *p* < 0.0001 when compared with the PBS control. ^#^ *p* < 0.05, ^##^ *p* < 0.01, and ^####^ *p* < 0.0001 when compared with FAC. DFCH-DA: 2′,7′-dichlorodihydrofluorescein diacetate; FI: fluorescence intensity; FAC: ferric ammonium citrate; DFP: deferiprone; GTE: green tea extract.

Malondialdehyde (MDA) is a lipid peroxidation product and major indicator of oxidative cellular damage induced by ROS [17]. In our research, iron overload dramatically elevated MDA levels in MG-63 cells (*p* < 0.0001) compared with the PBS group, indicating that excess iron promotes extensive lipid peroxidation (Figure 3a). Vitamin E, a powerful antioxidant, served as a positive control and significantly reduced MDA levels [18]. Similarly, treatment with DFP, GTE, and their combination dramatically reduced MDA levels in iron-overloaded MG-63 cells, with more pronounced effects observed at higher concentrations. Among these treatments, the combination therapy with a 10 µM concentration showed a superior reduction in lipid peroxidation, comparable with that of vitamin E and more effective than either DFP or GTE alone.

DCFH-DA (2′,7′-dichlorodihydrofluorescein diacetate) is a cell-permeable dye that is oxidized by ROS within cells to form the fluorescent compound 2′,7′-dichlorofluorescein (DCF) [19]. As shown in Figure 3b,c, FAC-induced cells exhibited a significant increase in FI (*p* < 0.01) compared with the non-iron-loading (PBS) control, signifying that excess iron heightened intracellular ROS levels. Thereafter, FI was markedly reduced following the DFP treatment. Consistently, GTE demonstrated notable antioxidant properties by reducing FI in a concentration-dependent manner. The combination therapy of DFP and GTE at 10 µM significantly reduced intracellular fluorescence (*p* < 0.01), with an effect comparable to DFP monotherapy.

### 3.4. Bone Formation and Mineralization Affected by GTE

**Figure 4:**
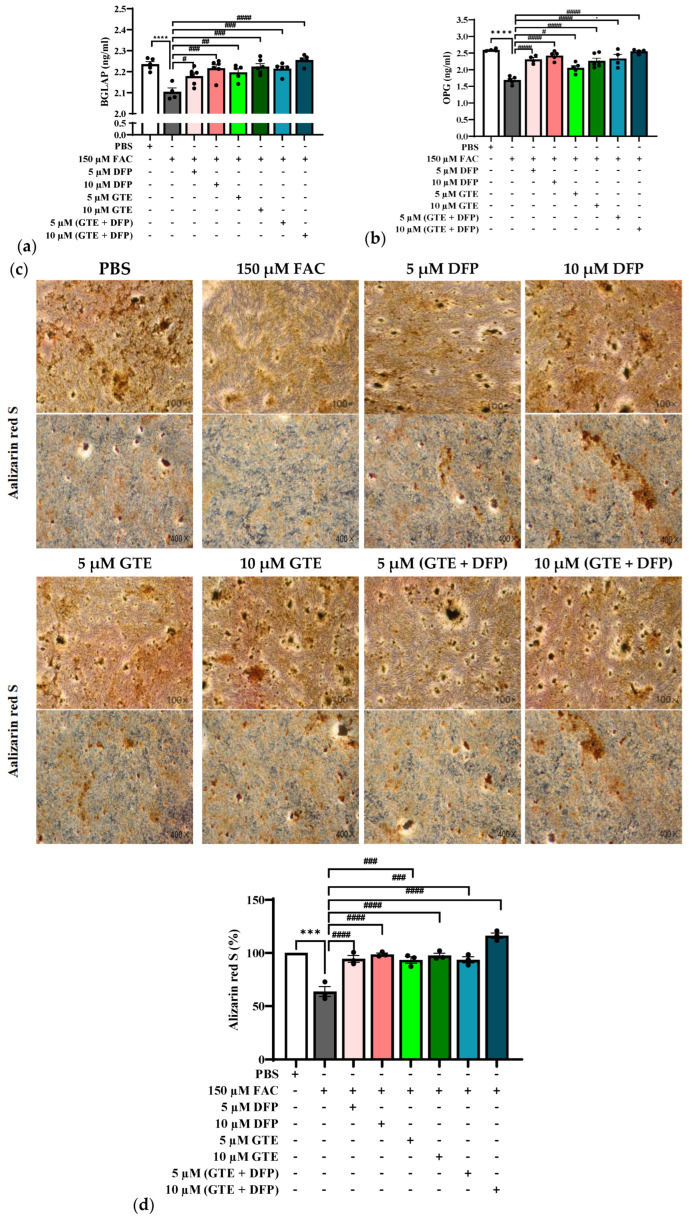
Effects of GTE, DFP, and their combination on bone formation markers’ mineralization in iron-loaded and non-iron-loaded MG-63 cells: BGLAP (**a**) and OPG (**b**). Bone mineralization and bone nodules were observed and photographed with a microscope (under 100× and 400× magnification) (**c**). Statistical results for the percentage of bone mineralization (**d**). Data are expressed as mean ± SEM (*n* = 3). Accordingly, *** *p* < 0.01 and **** *p* < 0.0001 when compared with the PBS control. ^#^ *p* < 0.05, ^##^ *p* < 0.01, ^###^ *p* < 0.001, and ^####^ *p* < 0.0001 when compared with FAC. BGLAP: bone gamma-carboxyglutamate protein; OPG: osteoprotegerin; FAC: ferric ammonium citrate; DFP: deferiprone; GTE: green tea extract.

Bone gamma-carboxyglutamate protein (BGLAP, or osteocalcin) is crucial in bone mineralization and matrix maturation [20], while osteoprotegerin (OPG) facilitates bone formation by inhibiting osteoclast-mediated bone resorption [21]. Figure 4a,b show that the expression levels of both BGLAP and OPG significantly reduced (*p* < 0.0001) following 6 h of FAC induced in MG-63 cells, suggesting that excessive iron has a negative impact on bone formation. However, treatment with 5 µM and 10 µM DFP for 8 h greatly improved BGLAP and OPG expressions, with the most pronounced effect observed at 10 µM (*p* < 0.001 for BGLAP; *p* < 0.0001 for OPG). The results suggest that the iron chelator plays a critical role in preserving bone formation markers by reducing intracellular iron levels. Similarly, the treatment with GTE (which contained equivalent EGCG) dramatically increased BGLAP (*p* < 0.01 for 5 µM; *p* < 0.001 for 10 µM) and OPG (*p* < 0.05 for 5 µM; *p* < 0.0001 for 10 µM) levels in iron-overloaded MG-63 cells, demonstrating a dose-dependent manner. Notably, the GTE treatment led to a greater enhancement in the BGLAP expression compared with DFP, suggesting that GTE may exert a stronger stimulatory effect on this marker. Although the combination of DFP and GTE elevated both BGLAP and OPG levels, it did not produce a superior effect compared with the individual therapy.

To confirm the bone cell mineralization of each group, Alizarin red S staining was applied to all groups (Figure 4c). After 6 h of FAC-induced iron overload, bone mineralization was significantly reduced (*p* < 0.0001) compared with the PBS group, which was represented by the disappearance of the brown nodules. The bone mineralization was accompanied by the disappearance of bone-mineralized nodules, indicating the disruption of the mineralization process during an excess of iron. However, the mineralization was significantly restored (*p* > 0.0001) after 8 h of treatment with 5 µM and 10 µM DFP. Consistently, the treatment with GTE led to a notable increase in bone mineralization in the iron-overloaded cell model (*p* < 0.001 for 5 µM; *p* < 0.0001 for 10 µM) in a dose-dependent manner (Figure 4d). Remarkably, 10 µM of the GTE and DFP combination therapy exhibited the greatest mineralization compared with either alone, indicating that a combined therapy supports bone matrix mineralization.

## 4. Discussion

Bone loss is a common complication associated with β-thalassemia, primarily resulting from iron overload due to ineffective erythropoiesis, heightened iron absorption, and systemic iron overload [18]. Excess iron accumulates in various organs, including bone tissue, where it contributes to the generation of ROS and induces oxidative stress, thereby disrupting bone remodeling. This ultimately leads to bone loss and an increase in the risk of osteoporosis [22]. Iron-chelating agents are widely used to manage iron overload in patients with β-thalassemia. DFP, as a commonly recommended chelator in medical clinics, has exhibited excellent iron-chelating and antioxidant capabilities [23]. Nonetheless, its application is frequently subject to serious side effects, including joint symptoms such as pain, stiffness, and arthritis [24,25]. The adverse effects might be aggravated and troubling for patients with hematological disorders and osteoporosis. Therefore, identifying safer, plant-derived alternatives with both iron-chelating and bone-protective properties is a major objective of our research. In this study, we explored the effects of GTE (which contained equivalent EGCG) and DFP on bone formation and mineralization of iron-overloaded MG-63 cells.

Excessive iron deposition in bone tissue disrupts normal bone homeostasis by interfering with osteoblast function and enhancing osteoclast activity, ultimately resulting in bone loss [5]. A clinical study revealed iron overload in the bones of patients with hematological disorders, resulting in compromised bone remodeling and increased fracture risk [26]. In this study, intracellular iron content measurements and fluorescence microscopy demonstrated that treatments with GTE and DFP significantly reduced intracellular iron levels. Notably, GTE alone or in combination with DFP exhibited a continued gradual decline in intracellular iron content beyond the plateau phase, suggesting that GTE can induce a slow and stable reduction in intracellular iron levels compared with DFP alone over time. These results align with previous studies on the iron-chelating capacity of GTE in various organs such as the liver and brain, where it reduces iron deposition and alleviates iron-induced toxicity through its iron-binding properties [27,28]. The ability of GTE to effectively chelate iron highlights its potential as a natural therapeutic agent for managing bone complications associated with iron-overload conditions.

Iron overload has been clarified to exacerbate osteoporotic conditions by elevating ROS production and inducing mitochondrial dysfunction, ultimately impairing osteoblast function and bone mineralization [29]. Our results are further supported by the fact that FAC markedly increased intracellular ROS and malondialdehyde (MDA) levels in osteoblast-like MG-63 cells, resulting in oxidative-stress-induced cellular damage. Green tea has been widely utilized as a polyphenolic material owing to its antioxidant properties and its ability to regulate various cellular activities [30]. In our research, GTE significantly reduced both MDA levels and ROS production in MG-63 cells, indicating an effect of GTE by mitigating iron-induced oxidative stress through chelating excess iron and suppressing iron-catalyzed free radical formation. Previous studies have demonstrated the positive effects of GTE against iron-induced oxidative damage in several organs, such as the liver, heart, and kidneys [31]. Capasso L. reported that EGCG reduces iron accumulation along with oxidative stress in cardiovascular diseases and metabolic syndromes, highlighting its antioxidant properties [32]. In addition, Yoshiomi O et al. confirmed that EGCG inhibits osteoclast formation and differentiation in rats [33]. Collectively, these studies highlight the multifaceted role of GTE in alleviating iron-induced oxidative damage, not only in systemic organs such as the liver and heart, but also in bone tissue, contributing to the maintenance of bone formation under iron-overload conditions.

Bone formation and mineralization are tightly regulated by key markers, with osteoprotegerin (OPG) and bone gamma-carboxyglutamate protein (BGLAP, or osteocalcin) serving as crucial indicators of these processes. OPG functions as a decoy receptor for the receptor activator of nuclear factor kappa-B ligand (RANKL), thereby obstructing osteoclast development and facilitating bone growth [34]. BGLAP contributes to bone matrix maturation by binding to calcium and hydroxyapatite, the primary mineral components of bone tissue [35]. Our results reveal that iron overload notably downregulated the expression of both OPG and BGLAP, suggesting that elevated iron levels interfere with bone formation and remodeling processes. Treatments with DFP and GTE resulted in substantial elevations in OPG and BGLAP levels, exhibiting a concentration-dependent effect. These results are consistent with previous studies reporting that polyphenols can prompt osteoblast differentiation and bone matrix synthesis [36].

Bone mineralization is a crucial outcome of osteoblastic activity and an essential indicator of skeletal health [37]; this was also markedly suppressed by iron overload, as demonstrated by Alizarin red S staining, which revealed a reduction in mineralized nodules. This observation aligns with prior findings indicating that excess iron induces bone loss through oxidative damage [3]. Treatment with both DFP and GTE greatly reestablished mineralization in a dose-dependent manner. The ability of GTE to improve bone mineralization may be attributed to its dual function in reducing iron deposition and oxidative stress.

Interestingly, the combination of GTE and DFP revealed bone formation and mineralization effects, even though the effect of the combination was not significantly different from the individual treatment. This may have been due to their overlapping iron-chelation mechanisms, as both primarily bind to free iron, leading to a saturation effect. Differences in bioavailability and cellular uptake mechanisms may potentially account for this outcome. However, the natural properties of GTE offer significant advantages and safety, thereby opening new therapeutic possibilities.

Although our in vitro findings demonstrate the potential of green tea extract (GTE) to mitigate iron-induced oxidative stress and promote bone formation in MG-63 cells, these in vitro findings have limitations. The in vitro system lacks the physiological complexity of human systems, including bioavailability, metabolism, and patient-specific factors (e.g., age, comorbidities, or hormonal status). We acknowledge that experimental models significantly differ from clinical scenarios. In vivo factors such as bioavailability, metabolism, and patient-specific variables (e.g., age, comorbidities, and dietary patterns) may influence the efficacy and safety of GTE. Notably, studies from the InCHIANTI cohort, such as Pellegrino et al. [38] and Di Iorio et al. [39], have reported that a high intake of dietary polyphenol may be associated with increased risks of osteoporosis and fractures, potentially due to dose-dependent effects or interactions with bone metabolism pathways.

To support the clinical potential of GTE for bone health, several human studies have demonstrated its bone-protective effects. Wu et al. [40] found that habitual tea consumption (including green tea) over 10 years was associated with higher bone mineral density (BMD) in the lumbar spine and hips of 1037 men and women, with no increased fracture risk. Shen et al. [41] conducted a 6-month randomized, placebo-controlled trial with 171 postmenopausal women with osteopenia, and showed that GTE supplementation (500 mg/day) improved BMD at the proximal femur and reduced oxidative stress markers. Devine et al. [42] reported that tea consumption in 1500 older women was linked to higher BMD and lower fracture prevalence, suggesting green tea’s protective effects against osteoporosis. These clinical findings align with our results, indicating that moderate, controlled GTE intake can enhance bone health in populations at risk of bone loss, particularly where oxidative stress is a contributing factor, as in our iron-overload model.

These findings underscore the need for the cautious interpretation of our results as well as further clinical studies to determine optimal GTE dosing and its long-term effects on bone health in patients with iron-overload conditions.

## 5. Conclusions

In summary, this study demonstrates that green tea extract (GTE) enriched with epigallocatechin-3-gallate (EGCG) effectively attenuates iron-induced oxidative stress and promotes osteogenic activity, including bone formation and mineralization, in iron-overloaded MG-63 osteoblast-like cells. Furthermore, the natural origin and favorable safety profile of GTE suggest its potential as a supportive strategy for patients experiencing bone-related complications associated with long-term iron-chelation therapy. However, the translation of these in vitro findings to clinical settings requires further investigation, particularly to address the optimal dosage associated with safety.

## Data Availability

The data that support the findings of this study are available from the corresponding author upon reasonable request.

## Supplementary Materials

The following supporting information can be downloaded at https://www.mdpi.com/article/10.3390/antiox14070874/s1. Figure S1: High-performance liquid chromatography analysis of EGCG content in GTE (5 mg/mL).

## Abbreviations

The following abbreviations are used in this manuscript:

ANOVA	Analysis of variance
BGLAP	Bone gamma-carboxyglutamate protein
C	Catechin
DCF	2′,7′-Dichlorodihydrofluorescein
DCFH-DA	2′,7′-Dichlorodihydrofluorescein diacetate
DFP	Deferiprone
DMEM	Dulbecco’s Modified Eagle Medium
ECG	Epicatechin-3-gallate
EGC	Epigallocatechin
EGCG	Epigallocatechin-3-gallate
ELISA	Enzyme-linked immunosorbent assay
FAC	Ferric ammonium citrate
FBS	Fetal bovine serum
FI	Fluorescence intensity
GTE	Green tea extract
MDA	Malondialdehyde
MG-63	Human osteoblast-like cells
MPA	Meta-phosphoric acid
MTT	3-(4,5-Dimethylthiazol-2-yl)-2,5-diphenyl tetrazolium bromide
OPG	Osteoprotegerin
RANKL	Receptor activator of nuclear factor kappa-B ligand
ROS	Reactive oxygen species
SEM	Standard error of the mean
TBA	Thiobarbituric acid
