# Supplementary figures and images for: Green Tea Extract Containing Epigallocatechin-3-Gallate Facilitates Bone Formation and Mineralization by Alleviating Iron-Overload-Induced Oxidative Stress in Human Osteoblast-like (MG-63) Cells
Source: Antioxidants (Basel). 2025 Jul 17;14(7):874. doi: 10.3390/antiox14070874 (PMC12292158; doi:10.3390/antiox14070874)

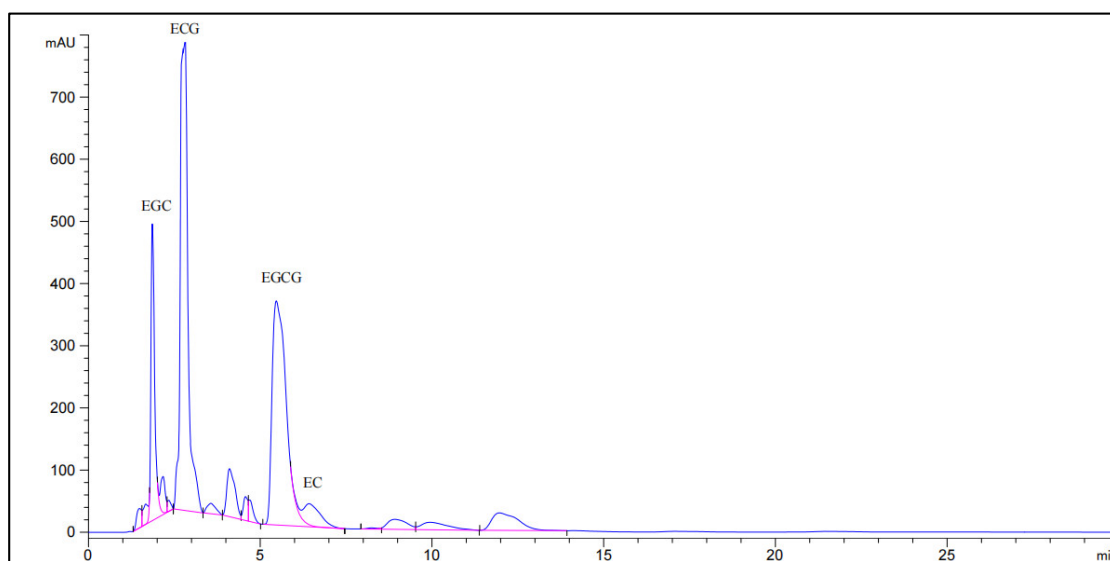

**Figure S1.** High-performance liquid chromatography analysis of EGCG content in GTE (5 mg/mL).

Supplement: Supplementary file 1 [file antioxidants-14-00874-s001.zip › antioxidants-3730287-supplementary.pdf]
